# RNase A Treatment Interferes With Leukocyte Recruitment, Neutrophil Extracellular Trap Formation, and Angiogenesis in Ischemic Muscle Tissue

**DOI:** 10.3389/fphys.2020.576736

**Published:** 2020-11-06

**Authors:** Manuel Lasch, Konda Kumaraswami, Simona Nasiscionyte, Susanna Kircher, Dominic van den Heuvel, Sarah Meister, Hellen Ishikawa-Ankerhold, Elisabeth Deindl

**Affiliations:** ^1^Walter-Brendel-Centre of Experimental Medicine, University Hospital, Ludwig-Maximilians-Universität München, Munich, Germany; ^2^Biomedical Center, Institute of Cardiovascular Physiology and Pathophysiology, Ludwig-Maximilians-Universität München, Munich, Germany; ^3^Department of Otorhinolaryngology, Head and Neck Surgery, University Hospital, Ludwig-Maximilians-Universität München, Munich, Germany; ^4^Department of Obstetrics and Gynaecology, University Hospital, Ludwig-Maximilians-Universität München, Munich, Germany; ^5^Department of Internal Medicine I, Faculty of Medicine, University Hospital, Ludwig-Maximilians-Universität München, Munich, Germany

**Keywords:** angiogenesis, capillary sprouting, RNase A, RNase 5, extracellular RNA, leukocyte recruitment, ischemia, neutrophil extracellular traps

## Abstract

**Background:** RNase A (the bovine equivalent to human RNase 1) and RNase 5 (angiogenin) are two closely related ribonucleases. RNase 5 is described as a powerful angiogenic factor. Whether RNase A shares the same angiogenic characteristic, or interferes with vessel growth as demonstrated for arteriogenesis, has never been investigated and is the topic of this present study.

**Methods and Results:** To investigate whether RNase A shows a pro‐ or anti-angiogenic effect, we employed a murine hindlimb model, in which femoral artery ligation (FAL) results in arteriogenesis in the upper leg, and, due to provoked ischemia, in angiogenesis in the lower leg. C57BL/6J male mice underwent unilateral FAL, whereas the contralateral leg was sham operated. Two and seven days after the surgery and intravenous injection of RNase A (50 μg/kg dissolved in saline) or saline (control), the gastrocnemius muscles of mice were isolated from the lower legs for (immuno-) histological analyses. Hematoxylin and Eosin staining evidenced that RNase A treatment resulted in a higher degree of ischemic tissue damage. This was, however, associated with reduced angiogenesis, as evidenced by a reduced capillary/muscle fiber ratio. Moreover, RNase A treatment was associated with a significant reduction in leukocyte infiltration as shown by CD45^+^ (pan-leukocyte marker), Ly6G^+^ or MPO^+^ (neutrophils), MPO^+^/CitH_3_^+^ [neutrophil extracellular traps (NETs)], and CD68^+^ (macrophages) staining. CD68/MRC1 double staining revealed that RNase A treated mice showed a reduced percentage of M1-like polarized (CD68^+^/MRC1^−^) macrophages whereas the percentage of M2-like polarized (CD68^+^/MRC1^+^) macrophages was increased.

**Conclusion:** In contrast to RNase 5, RNase A interferes with angiogenesis, which is linked to reduced leukocyte infiltration and NET formation.

## Introduction

The human superfamily of ribonucleases (RNases) consists of eight members of secreted proteins, which share the ability to degrade RNA (Koczera et al., 2016). RNase A (the bovine equivalent to the human RNase 1) is one of the best-characterized mammalian proteins (Raines, 1998). According to their structural and biological or catalytic characteristics, the RNases have been grouped into four RNase types, whereby RNase 1, 4, and 5 have been grouped together (Sorrentino, 2010). RNase 5 shows high similarities to RNase A in its crystal structure and contains several identical catalytic residues, although its ribonucleolytic activity is much lower (Acharya et al., 1994; Leonidas et al., 1999; Leland et al., 2002).

Studies on their molecular evolution indicated that ancestral RNases were involved in host defense (zebrafish) or angiogenesis-related processes (birds and mammals; Sorrentino, 2010; Koczera et al., 2016). Interestingly, RNase 1 has been suggested to be involved in the regulation of vascular homeostasis (Landre et al., 2002; Fischer et al., 2011) and has been demonstrated to negatively influence coagulation and vascular permeability (Fischer et al., 2007; Kannemeier et al., 2007). Both, RNase 1 and RNase 5 are expressed and released from various types of endothelial cells (from arteries, veins, and capillaries; Landre et al., 2002; Fischer et al., 2011), and RNase 5, also termed angiogenin, is well described for its angiogenic activity (Fett et al., 1985; Strydom et al., 1985) as reviewed in detail by Gao and Xu (2008) as well as Sorrentino (2010). By complex formation with the cell surface protein actin, RNase 5 promotes basement membrane and extracellular matrix degradation, thus enabling endothelial cells to penetrate and migrate into the perivascular tissue (Soncin, 1992). Following translocation to the nucleus, the nuclear fraction of RNase 5 binds to the promoter of ribosomal RNA (rRNA), thereby enhancing its transcription and promoting endothelial cell proliferation (Kishimoto et al., 2005). In addition to its function to enhance angiogenesis, RNase 5 has also been reported to cleave transfer RNA (tRNA) to tRNA-derived small RNAs (tiRNAs) under conditions of stress, causing protein synthesis arrest, thus conserving energy for repair of damaged tissue (Fu et al., 2009; Yamasaki et al., 2009).

Angiogenesis is a complex process resulting in increased capillarity. This is either mediated by splitting of pre-existing capillaries (Egginton et al., 2001) or by sprouting of capillaries from pre-existing vasculature involving endothelial cell proliferation and migration (Carmeliet, 2000). Hypoxia and ischemia are well described as driving force for sprouting angiogenesis, however, there are also data available pointing to shear stress as stimulus for splitting angiogenesis (Egginton et al., 2001). In general, angiogenesis is designed to locally satisfy the oxygen and nutrient demand of tissue under various (patho-)physiological conditions (Adams and Alitalo, 2007; Potente and Carmeliet, 2017) such as embryonic development (Carmeliet, 2005), tumor growth (Folkman, 1995), wound healing (Tonnesen et al., 2000), or skeletal or cardiac muscle hypertrophy (Hudlicka et al., 1992). However, in damaged ischemic tissue, it is also engaged in removal of cell debris (Weckbach et al., 2018).

Vascular endothelial growth factor (VEGF) is one of the strongest and best characterized angiogenetic factors (Folkman, 1971; Folkman et al., 1971; Ferrara and Henzel, 1989; Neufeld et al., 1999). It has been demonstrated that administration of VEGF is a powerful tool to promote sprouting angiogenesis *in vivo* and even results in angioma formation (Isner et al., 1996; Tsurumi et al., 1997; Schwarz et al., 2000). Arteriogenesis, the growth of a natural bypass from pre-existing arteriolar connections (Deindl and Schaper, 2005), however, could hardly if at all be promoted by VEGF administration (Jazwa et al., 2016). On the other hand, blocking the tyrosine kinase (TK) VEGF receptor 2 (VEGFR2) strongly interfered with both, the process of angiogenesis and arteriogenesis (Jazwa et al., 2016). In both types of vessel growth, VEGF is supplied by leukocytes, i.e., neutrophils and monocytes (Deindl et al., 2001; Scapini et al., 2004; Morrison et al., 2014; Lautz et al., 2018; Zhang et al., 2020). But in contrast to angiogenesis, in arteriogenesis, an amplified and sustained local activation of VEGFR2 is necessary to promote endothelial cell proliferation by high levels of ERK activation (Kofler and Simons, 2015). The sustained activation of VEGFR2 is mediated by the non-TK VEGF receptor neuropilin 1 (NRP1; Koch et al., 2011; Lanahan et al., 2013; Kofler and Simons, 2015). Recently, it was demonstrated by Fischer et al. (2009) *in vitro* that the interaction of VEGF with VEGFR2 and NRP1 is mediated by extracellular RNA. For angiogenesis, it is described that the interaction of VEGF with VEGFR2 and NRP1 is essential for tip cell formation to allow a sprouting of endothelial cells (Kofler and Simons, 2015).

RNA, which is mainly composed of rRNA is released under various conditions, such as tissue damage, stress, and accordingly also increased shear stress from (endothelial-) cells (Lasch et al., 2019a). Recently, we demonstrated that arteriogenesis, which is triggered by increased shear stress (Pipp et al., 2004; Lasch et al., 2019b), is initiated by extracellular RNA, which is released from endothelial cells due to increased shear stress (Lasch et al., 2019a). In arteriogenesis, extracellular RNA induces a sustained activation of VEGFR2 provoking the release of von Willebrand factor (vWF) from endothelial cells. This initiates a cascade of signaling events, which involves an activation of platelets and mast cells. As a result, neutrophils and monocytes are recruited, which promote vessel growth by supplying growth factor and cytokines (Chandraratne et al., 2015; Chillo et al., 2016; Kluever et al., 2019; Lasch et al., 2019a). Interestingly, administration of bovine RNase A as well as recombinant human RNase 1 significantly interfered with the process of arteriogenesis and leukocyte recruitment, whereas the administration of inactive human recombinant RNase or DNase had no effect pointing to the relevance of extracellular RNA in arteriogenesis (Lasch et al., 2019a).

**Figure 1:**
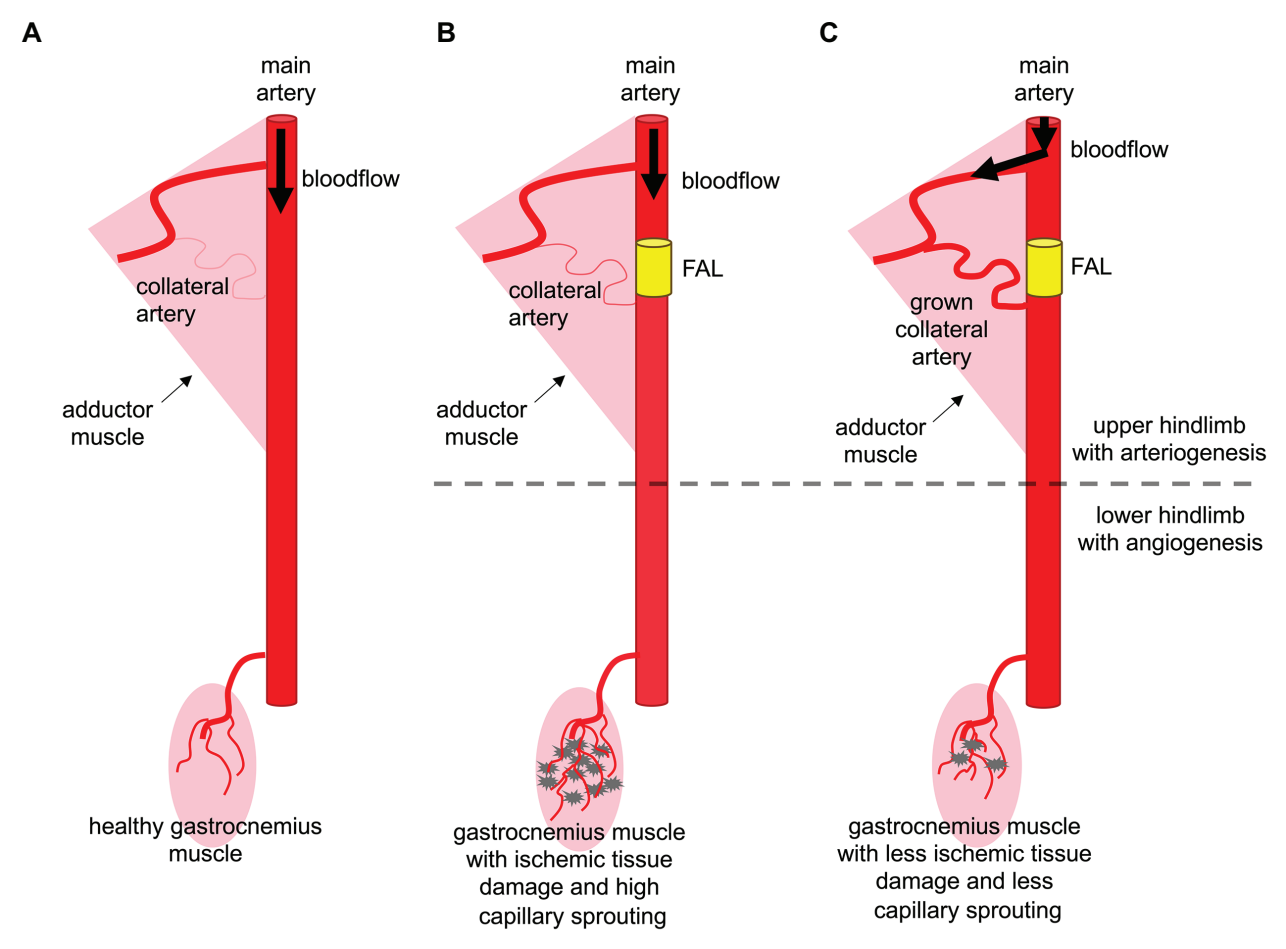
Arteriogenesis in the thigh protects the gastrocnemius muscle from serve ischemic damage. **(A)** Arterial blood vessels have the function to supply organs and muscle tissue with oxygen and nutrients. In the murine hindlimb, the femoral artery supplies blood to the lower leg. **(B)** Upon arterial obstruction due to stenosis or artificial femoral artery ligation (FAL), muscles of the lower leg, such as the gastrocnemius muscle, suffer from ischemia, which leads to tissue damage and capillary sprouting (angiogenesis). **(C)** Effective collateral artery growth (arteriogenesis) in the thigh compensating the occluded (femoral) artery prevents severe ischemic tissue damage in the lower leg and makes capillary sprouting redundant.

RNase A and RNase 5 are two closely related ribonucleases (Gao and Xu, 2008; Sorrentino, 2010). Accordingly, we were interested to investigate whether RNase A shows a similar pro-angiogenic effect as previously described for RNase 5 (Gao and Xu, 2008), or whether it interferes with the process of angiogenesis as demonstrated for arteriogenesis (Lasch et al., 2019a). For our purpose, we used the same mouse model, which we employed to investigate the effect of RNase A treatment on arteriogenesis (Lasch et al., 2019a). In that model, femoral artery ligation (FAL) results due to increased shear stress in arteriogenesis in the upper leg and due to ischemia in angiogenesis in the lower leg (Figure 1). Whether shear stress is also involved in promoting angiogenesis in that particular animal model has never been investigated but cannot be excluded.

## Materials and Methods

### Animals and Treatments

All experimental procedures were permitted by the Bavarian Animal Care and Use Committee (ethical approval code: ROB-55.2Vet-2532.Vet_02-17-99) and were done in strict accordance with the German animal legislation guidelines. To degrade extracellular RNA, wild type C57BL/6J mice (Charles River Laboratories, Sulzfeld, Germany) were injected i.v. with bovine RNase A (Thermo Fisher Scientific, Waltham, MA, United States) with a dose of 50 μg/kg dissolved in saline starting 30 min before the surgical procedure and followed every other day until tissue sampling. The control group received saline alone.

### Femoral Artery Ligation and Tissue Sampling

To induce angiogenesis, 8–10 weeks old mice were initially anesthetized with a combination of fentanyl (0.05 mg/kg, CuraMED Pharma, Karlsruhe, Germany), midazolam (5.0 mg/kg, Ratiopharm GmbH, Ulm, Germany), and medetomidine (0.5 mg/kg, Pfister Pharma, Berlin, Germany). Once anesthetized, the mice were submitted to an unilateral FAL of the right hindlimb, while the left hindlimb was sham operated as previously described (Limbourg et al., 2009; Lasch et al., 2019b). For tissue sampling 2 or 7 days after the surgical procedure, mice were anesthetized as described above and were perfused with adenosine buffer [1% adenosine (Sigma-Aldrich, St. Louis, MO, United States), 5% bovine serum albumin (BSA, Sigma-Aldrich), dissolved in phosphate buffered saline (PBS, PAN Biotech, Aidenbach, Germany, pH 7.4)] followed by a perfusion with 3% paraformaldehyde (PFA, Merck, Darmstadt, Germany; for cryopreservation), or 4% PFA (for paraffin embedding) in PBS, pH 7.4. After the perfusion, the gastrocnemius muscles were carefully isolated and stored for further processing.

### Histology and Immunohistology

**Figure 2:**
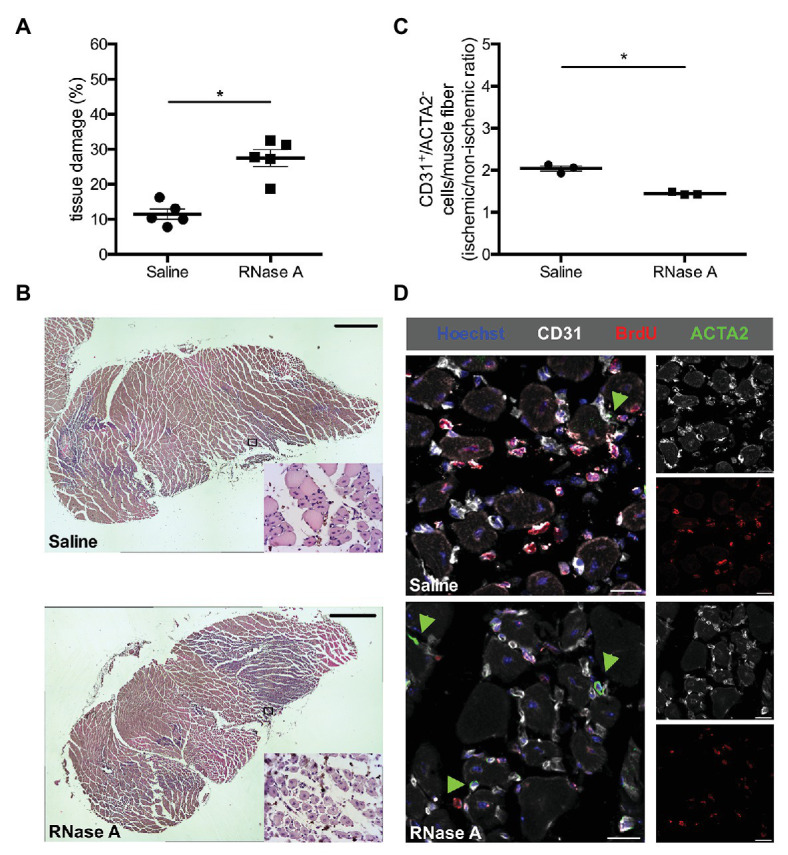
RNase A treatment results in decreased angiogenesis and increased tissue damage. **(A)** The scatter plot shows the percentage of tissue damage in the gastrocnemius muscle of mice treated with RNase A or saline (control group) 7 days after FAL. Data are means ± S.E.M., *n* = 5 per group, the whole cross-sectional area of the gastrocnemius muscle was analyzed per mouse. ^*^p < 0.05 (RNase A vs. saline treated group) by unpaired student’s *t*-test. **(B)** Representative pictures of analyzed H and E stained gastrocnemius muscle of saline‐ (upper picture) or RNase A (lower picture) treated mice 7 days after FAL. After treatment with bovine RNase A, significant increased tissue damage can be seen becoming evident e.g., by skeletal muscle cells showing centralized nuclei (small magnifications right bottom). Scale bars 100 μm. **(C)** The scatter plot shows the CD31^+^/ACTA2^−^ cells per muscle fiber (ischemic vs. non-ischemic tissue ratio, respectively) in the gastrocnemius muscle of RNase A or saline treated mice 7 days after the surgical procedure. Data are means ± S.E.M., *n* = 3 per group, 10 cross-sectional areas (450 μm × 450 μm each) of the gastrocnemius muscle were analyzed per mouse per leg. ^*^p < 0.05 (RNase A vs. saline treated group) by unpaired student’s *t*-test. **(D)** Representative immunofluorescence pictures of analyzed gastrocnemius muscle tissue of saline‐ (upper picture) or RNase A treatment (lower picture) 7 days after FAL. Endothelial cells were labeled with anti-CD31 (gray), with anti-BrdU 546 (red), and Hoechst (blue). Pericytes, in addition, were labeled with anti-ACTA2 (green, and indicated by green arrowheads). Scale bars 20 μm.

Mice (*n* ≥ 3 per group) were injected (i.p.) daily with BrdU (Sigma-Aldrich; 1.25 mg dissolved in 100 μl PBS) starting directly after FAL. BrdU-treated tissue isolated at day 7 after the surgical procedure was cut in 8–10 μm thick cryosections and was processed with 1 N HCl for 30 min at 37°C, blocked with 10% goat serum in 1x PBS/0.1% Tween-20 for 1 h at room temperature (RT), followed by an overnight incubation with an anti-BrdU antibody (Abcam, ab6326; dilution 1:50 in 10% goat serum in 1x PBS/0.1% Tween-20) at 4°C. Secondary staining was performed with a goat anti-rat Alexa Fluor®-546 antibody or an Alexa Fluor®-647 antibody (both Invitrogen, Thermo Fisher Scientific, dilution 1:100) for 1 h at RT. Further, the tissues were stained with an anti-CD31-Alexa Fluor®-647 antibody (1:50; clone MEC13.3, Biolegend, 102,516) or an anti-CD45-Alexa Fluor®-488 antibody (clone 30-F11, BioLegend, 11-0451-85; dilution 1:100, both in 1x PBS/0.1% Tween-20) for 3 h at RT to stain endothelial cells and leukocytes, respectively. Pericytes were stained with an anti-ACTA2-Alexa Fluor®-488 antibody (Sigma-Aldrich, F3777) 1:400 dilution in 1x PBS/0.1% Tween-20. For neutrophil labeling, a rat anti-Ly6G antibody (Abcam, ab25377; 1:100) was added overnight at 4°C and a secondary Alexa Fluor®-488 goat anti-rat antibody (Invitrogen, Thermo Fisher Scientific) 1:200 in 1x PBS for 1 h. We have also followed this staining with an anti-Ly6G-PE antibody (eBioscience, 12-9668-82, Thermo Fisher Scientific) to eliminate any unspecific labeling. Macrophages were labeled with an anti-CD68-Alexa Fluor®-488 antibody (Abcam ab201844; dilution 1:50) together with an anti-MRC1 antibody (Abcam, ab64693; dilution 1:50) overnight 4°C, followed by the secondary antibodies: Alexa Fluor®-488 goat anti-rat and Alexa-Fluor®-594 goat anti-rabbit, respectively (both Invitrogen, Thermo Fisher Scientific). Neutrophil extracellular traps (NETs) of day 2 post-FAL tissues were labeled with an anti-myeloperoxidase (MPO) antibody (R and D Systems, AF3667) and an anti-citrullinated histone H3 antibody [Cit-H3; polyclonal rabbit anti-Histone H3 antibody (citrulline R2 + R8 + R17), Abcam, ab5103] by an overnight incubation at 4°C. This was followed by a secondary donkey anti-goat Alexa Fluor®-594 antibody (1:100) and Alexa Fluor®-488 antibody (1:200; both Invitrogen, Thermo Fisher Scientific) for 1 h at RT. Additionally, to label nucleic DNA, tissues were incubated with 1 μg/ml Hoechst 33342 (Invitrogen) for 15 min at RT. For mounting the samples, an antifade mounting medium (Dako) was used. Tissue muscle sections from saline and RNase A treated mice (3x saline treated non-ischemic, 3x saline treated ischemic, 3x RNase A treated non-ischemic, and 3x RNaseA treated ischemic) were stained for different leukocyte populations (neutrophils and macrophages) and NETs. We counted cells, muscle fibers, and NETs in 10 defined fields of view with a 20x objective (415 × 415 μm), resulting in a total area of 172,225 mm^2^. To investigate angiogenesis (Figure 2C), the capillary to muscle fiber ratio for each group was calculated as previously described (Olfert et al., 2016), whereby CD31^+^/ACTA2^−^ were considered as endothelial cells. The images were acquired with a confocal laser scanning microscope LSM 880 from (Carl Zeiss AG) and analyzed by ZEN Blue software (Carl Zeiss AG).

Hematoxylin and Eosin (H and E) staining on slices of 5 μm thick paraffin embedded gastrocnemius muscles isolated from RNase A and saline treated mice (*n* = 5) at day 7 after surgery was done according to standard procedures. The necrotic area (%) of the whole tissue slices was analyzed using an Axioscope 40 (Carl Zeiss AG) with the AxioVision software (Carl Zeiss AG).

### Statistical Analyses

Statistical analyses were carried out using GraphPad Prism 6 (GraphPad Software, La Jolla, CA, United States). Data are means ± standard error of the mean (S.E.M.). Statistical analyses were performed as indicated in the figure legends and considered as statistically significant at *p* < 0.05.

## Results

In order to investigate the impact of RNase A on angiogenesis, we used a murine hindlimb model, in which FAL results in arteriogenesis in the upper leg (adductor muscle) and due to provoked ischemia in angiogenesis in the lower leg (gastrocnemius muscle; Figure 1; Lasch et al., 2019a). Mice were treated intravenously before and then after the surgical procedure every other day with RNase A or saline (control). At day 2 and 7 after the surgical procedure, the gastrocnemius muscles of femoral artery ligated and contralateral sham operated legs were isolated for (immuno-) histological analyses.

Hematoxylin and Eosin staining demonstrated ischemic damage in gastrocnemius muscles of RNase A and of saline treated mice at day 7 after FAL. However, compared to the saline-treated controls, the ischemic tissue damage in gastrocnemius muscles was significantly increased in RNase A treated mice (Figures 2A,B and Supplementary Table 1).

To investigate whether RNase A treatment has an impact on angiogenesis, we performed immunofluorescence staining using an anti-CD31 antibody as a marker for endothelial cells, in combination with an antibody against ACTA2 to exclude CD31^+^ pericytes. Calculating the capillary/muscle fiber ratio as index for angiogenesis, we found a significant reduction of the capillary/muscle fiber ratio in gastrocnemius muscles of RNase A treated mice at day 7 after the surgical intervention when compared to saline treated control mice (Figures 2C,D and Supplementary Table 2). The number of proliferating endothelial cells per muscle fiber was also strongly reduced in RNase A treated mice as shown by CD31^+^/BrdU^+^/ACTA2^−^/Hoechst^+^ quadruple staining (Figure 2D; Supplementary Figure 1 and Supplementary Table 2).

**Figure 3:**
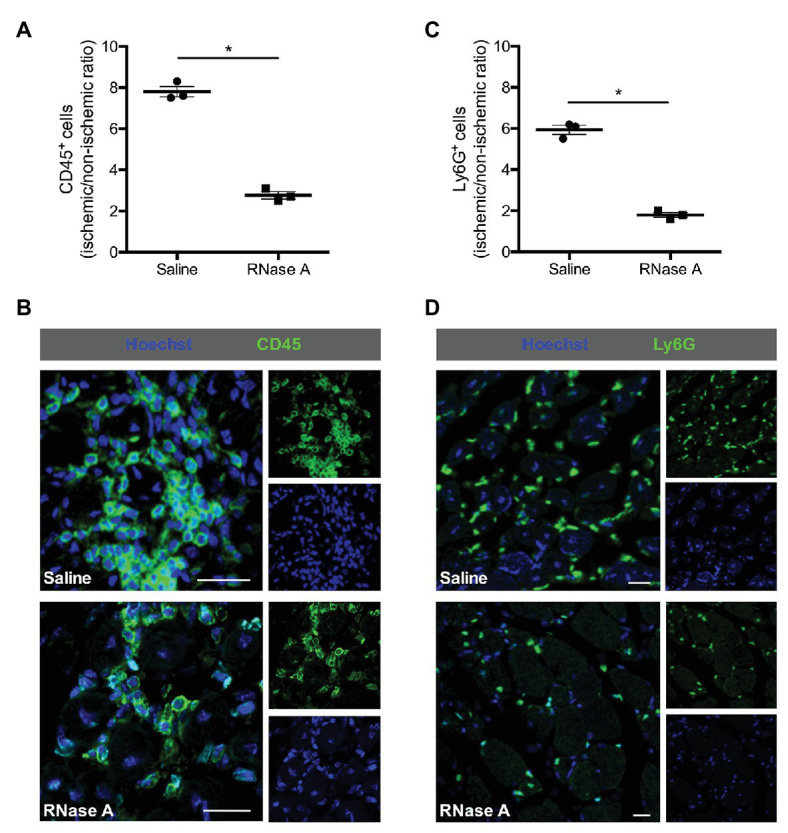
RNase A treatment interferes with leukocyte recruitment. The scatter plots show the ratio of **(A)** total CD45^+^ cells or **(C)** total Ly6G^+^ cells (neutrophils; FAL vs. sham operation or ischemic vs. non-ischemic tissue, respectively) in the gastrocnemius muscle of RNase A or saline treated mice 7 days after the surgery. Data are means ± S.E.M., *n* = 3 per group, 10 cross-sectional areas (450 μm × 450 μm each) of the gastrocnemius muscle were analyzed per mouse per leg. ^*^p < 0.05 (RNase A vs. saline treated group) by unpaired student’s *t*-test. **(B,D)** Representative immunofluorescence pictures of analyzed gastrocnemius tissue of saline‐ (upper picture) or RNase A (lower picture) treated mice 7 days after FAL. Leukocytes were labeled with **(B)** anti-CD45 (pan-leukocyte marker) or **(D)** anti-Ly6G (marker for neutrophils; both green) and Hoechst (blue). Scale bars 20 μm.

Leukocytes, such as neutrophils and macrophages, are well described for their relevance to remove cell debris and to promote angiogenesis by supplying growth factors, and particularly VEGF (Scapini et al., 2004; Zhang et al., 2020). To investigate the influence of RNase A treatment on leukocyte recruitment, we performed CD45 (pan-leukocyte marker) staining. Our results demonstrated that RNase A treated mice have a significant decreased number of CD45^+^ cells in ischemic tissue compared to saline treated control mice at day 7 after the surgical intervention (Figures 3A,B and Supplementary Table 2). In both treatments, only minor numbers of CD45^+^/BrdU^+^ double positive cells were found (Supplementary Figure 2).

**Figure 4:**
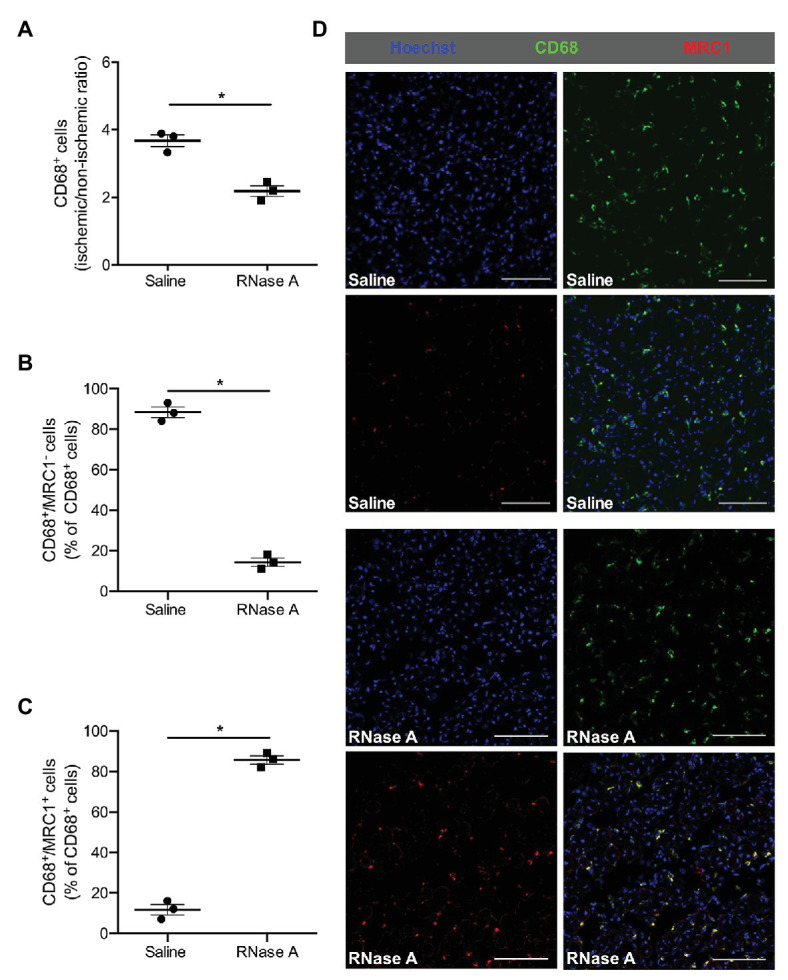
The effect of bovine RNase A on macrophage recruitment and polarization. The scatter plots show the ratio (ischemic vs. non-ischemic tissue) of **(A)** total CD68^+^ cells and the percentage of **(B)** CD68^+^/MRC1^−^ and of **(C)** CD68^+^/MRC1^+^ cells of all CD68^+^ cells in the gastrocnemius muscle of RNase A or saline treated mice 7 days after the surgical procedure. Data are means ± S.E.M., *n* = 3 per group, 10 cross-sectional areas (450 μm × 450 μm each) of the gastrocnemius muscle were analyzed per mouse per leg. ^*^p < 0.05 (RNase A vs. saline treated group) by unpaired student’s *t*-test. **(D)** Representative immunofluorescence pictures of analyzed gastrocnemius tissue of saline‐ (upper picture) or RNase A (lower picture) treated mice 7 days after FAL. Scale bars 100 μm.

Ly6G stain used to identify neutrophils and CD68 to identify macrophages showed moreover that both subsets of leukocytes were significantly decreased in ischemic muscle tissue of mice treated with RNase A compared to saline treated control mice at day 7 after the surgery (Figures 3C,D, 4A and Supplementary Table 2).

To investigate whether RNase A treatment influences macrophage activation in ischemic tissue in terms of M1-like and M2-like polarization, we performed CD68/MRC1 double staining. Our immuno-histological analyses showed that the percentage of classically activated inflammatory CD68^+^/MRC1^−^ macrophages (M1-like) in gastrocnemius muscles of RNase A treated mice was significantly decreased, while the percentage of alternatively activated regenerative CD68^+^/MRC1^+^ macrophages (M2-like) was significantly increased compared to saline treated control mice (Figures 4B–D and Supplementary Table 2).

**Figure 5:**
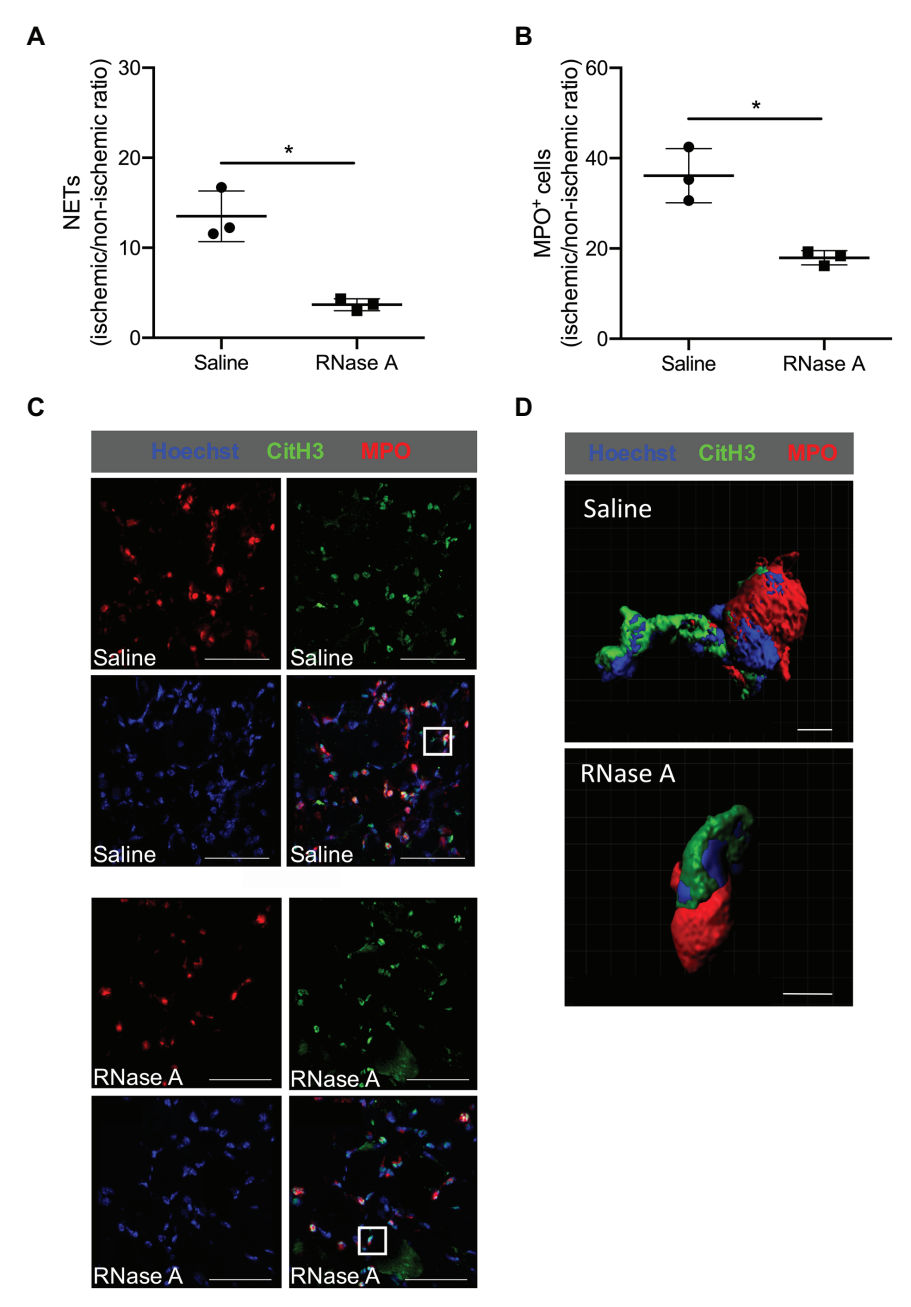
RNase A treatment results in reduced numbers of neutrophil extracellular traps (NETs). **(A,B)** The scatter plots show the ratio (ischemic vs. non-ischemic tissue) of NETs expression **(A)** or total MPO^+^ (myeloperoxidase) cells **(B)** of RNase A or saline treated mice 2 days after surgery. Data are means ± S.E.M., *n* = 3 per group, 10 cross-sectional areas (450 μm × 450 μm each) of the gastrocnemius muscle were analyzed per mouse per leg. ^*^p < 0.05 (RNase A vs. saline treated group) by unpaired student’s *t*-test. **(C)** Representative immunofluorescence pictures of analyzed gastrocnemius muscle tissue of saline‐ (upper picture) or RNase A (lower picture) treated mice 2 days after the surgery. NETs were stained with anti-MPO (red) and anti-CitH3 (green) and Hoechst (blue) for DNA staining. Scale bars 50 μm. **(D)** Representative immunofluorescence 3D reconstruction of a neutrophil with NET formation [magnification of white rectangle in **(C)**] of saline‐ (upper picture) or RNase A (lower picture) treated mice 2 days after FAL. Scale bars 4 μm.

In gastrocnemius muscle samples isolated at day 2 after the surgery and RNase A or saline treatment, we have used MPO staining for neutrophil identification combined with CitH3 for NETs. As a criteria for NETs, we have considered three aspects: (i) the presence of extracellular DNA filamentary structure, (ii) the DNA should originate from MPO positive cells, and (iii) these filamentary structures have to be decorated with a marker for neutrophil granule proteins like Cit-H3 (Figures 5C,D). As shown in Figure 5, the numbers of neutrophils (MPO^+^ cells) and NETs (MPO^+^/CitH3^+^/Hoechst^+^ cells) are reduced in ischemic gastrocnemius muscles from RNase A treated mice compared to saline treated mice (Figures 5A,B and Supplementary Table 2).

## Discussion

In the current study, we investigated the impact of RNase A, an enzyme closely related to RNase 5, on angiogenesis. Our results demonstrate that RNase A, in contrast to RNase 5, shows anti-angiogenic effects, which is related to reduced leukocyte recruitment and NET formation.

RNase A, like its human counterpart RNase 1 as well as RNase 5 belongs to the RNase A superfamily (Beintema, 1998). RNase 5 shows about 35% homology to the human RNase 1 with conserved amino acid residues relevant for the RNase activity (Kurachi et al., 1985). However, the ribonucleic activity of RNase 5 is about 10^5^–10^6^ lower than that of the pancreatic RNase A (Gao and Xu, 2008). It has previously been shown that RNase 5, also known as angiogenin, is a potent angiogenic factor (Kurachi et al., 1985). Whether RNase A shows similar functions in terms of promoting angiogenesis as RNase 5, or whether it interferes with vessel growth as we have recently demonstrated for the process of arteriogenesis (Lasch et al., 2019a), has never been investigated.

To analyze the impact of RNase A on angiogenesis, we employed the same murine hindlimb model, in which we have previously demonstrated that the administration of RNase A interferes with arteriogenesis by degrading extracellular RNA. Extracellular RNA is a ribonucleic acid, which is essential for initiating the process of natural bypass growth (Lasch et al., 2019a). As depicted in Figure 1, ligation of the femoral artery results in reduced blood supply and consequently causes tissue fibrosis and gangrene formation in the lower leg. This is associated with angiogenesis and a pronounced infiltration of leukocytes (Scapini et al., 2004; Jaipersad et al., 2014; Chillo et al., 2016; Zhang et al., 2020). In case of effective collateral artery growth, creating a natural bypass, a less severe ischemic tissue damage is observed in muscle tissue of the lower leg. However, in the case of impaired arteriogenesis, as shown before as an effect of RNase A (Lasch et al., 2019a), ischemic tissue damage is expected to increase. This was confirmed by our present histological studies, which demonstrate increased ischemic damage in the gastrocnemius muscle of mice treated with RNase A. Pancreatic RNase 1 has no cytotoxic side-effects (Gaur et al., 2001), and, although no receptors for RNases have been identified up to now (Schirrmann et al., 2009), these enzymes may indeed enter cells by endocytosis (Haigis and Raines, 2003). RNases, however, are quickly inactivated by the cytosolic RNase Inhibitor (Leland et al., 1998; Haigis et al., 2003), which binds extremely tightly to mammalian RNases and, thereby, blocks their catalytic activity (Leland et al., 1998; Leland and Raines, 2001; Haigis et al., 2003; Arnold and Ulbrich-Hofmann, 2006; Schirrmann et al., 2009). Moreover, we have recently demonstrated that RNase A application is not associated with any toxic side effects, even when 20-fold overdosed (Kleinert et al., 2016).

Increased ischemic damage is expected to be associated with increased angiogenesis (Chillo et al., 2016). However, our immuno-histological analyses showed that ischemic tissue damage in RNase A treated mice is associated with reduced angiogenesis along with reduced endothelial cell proliferation. Leukocytes, particularly neutrophils and monocytes, which are recruited to ischemic tissue, are an important source of VEGF, which is one of the most powerful angiogenic factors (Gong and Koh, 2010; Seignez and Phillipson, 2017; Zhang et al., 2020). In our study, we found a high number of leukocytes in ischemic damaged tissue, however, the number of BrdU positive and thus proliferating leukocytes was very low in saline as well as RNase A treated mice. This is in accordance with a recent study in a murine hindlimb model showing that not resident, proliferating leukocytes, but recruited leukocytes are responsible for increased numbers of immune cells in ischemic muscle tissue (Zhang et al., 2020). We show that in RNase A treated mice the number of leukocytes was significantly reduced in ischemic tissue when compared to saline treated mice indicating that RNase A treatment interferes with leukocyte recruitment to ischemic tissue, and thus may affect VEGF bioavailability.

Using a murine cremaster model of inflammation, we have recently shown by means of intravital microscopy analyses that extracellular RNA acts as pro-inflammatory factor by promoting leukocyte recruitment (Fischer et al., 2012). In the same model as well as in the murine hindlimb model of arteriogenesis, which we used for our current study on angiogenesis, we have demonstrated that extracellular RNA mediated leukocyte recruitment is dependent on VEGFR2 activation (Lasch et al., 2019a). In detail, extracellular RNA promotes the interaction of VEGF with NRP1 and VEGFR2, thus inducing a sustained local activation of VEGFR2, which results in vWF release from endothelial cells (Fischer et al., 2009; Lasch et al., 2019a). Subsequent platelet-neutrophil aggregate (PNA) formation promotes mast cell activation, which in turn results in neutrophil and monocyte recruitment (Chillo et al., 2016; Lasch et al., 2019a). Treatment of mice with RNase A, the natural counterpart of extracellular RNA, however, significantly interfered with leukocyte recruitment (Lasch et al., 2019a). In the current study, we found that RNase A treatment also impaired neutrophil and monocyte recruitment strongly suggesting that this was due to the degradation of extracellular RNA, which is released from cells due to ischemic tissue damage. Whether extracellular RNA mediated leukocyte recruitment in angiogenesis is dependent on the axis of VEGFR2-PNA mediated mast cell activation, or due to the previously described function of extracellular RNA to induce tumor necrosis factor (TNF)α release from monocytic cells involving TNF-α-converting enzyme (TACE) activation (Fischer et al., 2012), remains to be elucidated. However, the idea that extracellular RNA might activate mast cells *via* the VEGFR2 pathway in angiogenesis is intriguing, since mast cells are not only relevant for leukocyte recruitment but are also local sources of the angiogenic factors VEGF and RNase 5 (Hiromatsu and Toda, 2003; Kulka et al., 2009). Interestingly, it has recently been published that mast cells are important players in angiogenesis *in vivo*, as shown in a murine hindlimb model of ischemia (Bot et al., 2020).

The reduced accumulation of leukocytes in ischemic tissue of RNase A treated mice may, however, not only be a function of reduced leukocyte recruitment but also of reduced leukocyte extravasation. The expression of intracellular adhesion molecule 1 (ICAM1) on endothelial cells of post-capillary veins, which is a prerequisite for leukocyte transmigration and extravasation (Ley et al., 2007), is also a function of the VEGF/VEGFR2 system and thus might be dependent on the availability of extracellular RNA (Kluever et al., 2019).

Whether extracellular RNA might also be involved in VEGF induced NRP1-VEGFR2 mediated tip cell formation, thereby controlling capillary sprouting (Kofler and Simons, 2015) is another open question and remains to be addressed in further studies.

In a recent study, it has been shown that extracellular RNA is abundant in NETs in psoriatic skin and promotes in complex with LL37 further NET formation (Herster et al., 2020). In our study, we found that RNase A treatment was associated with reduced NET formation in ischemic tissue samples. However, this seems to be a direct consequence of reduced neutrophil infiltration, and not due to degradation of neutrophil-derived and LL37 complexed extracellular RNA, as LL37 protects extracellular RNA from degradation by RNase A (Tepekoylu et al., 2017; Herster et al., 2020). Interestingly, it has been demonstrated by another study that NETs promote capillary sprouting *in vitro* and *in vivo* (Aldabbous et al., 2016) and are relevant for reparative vascular regeneration in ischemic retina *in vivo* (Binet et al., 2020). Accordingly, RNase A treatment might interfere with angiogenesis by reducing the number of NETs.

Tissue ischemia is associated with an infiltration of monocytes, which mature to macrophages. Classically activated inflammatory M1-like (CD68^+^/MRC1^−^) macrophages are present in the beginning inflammatory phase and are involved in phagocytosis and further leukocyte recruitment. After the removal of necrotic tissue, M1-like macrophages repolarize toward alternatively activated regenerative M2-like (CD68^+^/MRC1^+^) macrophages and participate in tissue remodeling (Dort et al., 2019; Zhang et al., 2020). Our immuno-histochemical analyses evidenced that the percentage of regenerative M2 polarized macrophages in relation to the total number of macrophages was much higher in RNase A than in saline treated control mice. Saline treated mice on the other hand showed a very high percentage of inflammatory M1-like macrophages. *In vitro* studies have demonstrated that bone marrow-derived macrophages upon treatment with extracellular RNA are skewed toward M1-like macrophages and express increased levels of inflammatory cytokines such as TNFα and interleukin 6 (IL-6; Cabrera-Fuentes et al., 2015). Accordingly, RNase A treatment should interfere with the polarization of macrophages toward the M1-like phenotype and favor the switch toward M2-like polarization, as observed in the current study. In our study on arteriogenesis, using the same animal model, however, we found reduced numbers of CD68^+^/MRC1^−^ as well as CD68^+^/MRC1^+^ macrophages around growing collateral arteries in RNase A treated mice when compared to saline treated controls (Lasch et al., 2019a). Together, these data indicate that not only extracellular RNA but further micro-environmental conditions and factors control macrophage polarization. Interestingly, a recent study on a murine hindlimb model showed that lactate, a metabolite which is not found in increased amounts in tissue harboring growing collaterals (Deindl et al., 2001), is a major determinant of M2-like polarization of macrophages in ischemic muscle tissue (Zhang et al., 2020).

In summary, we show that RNase A counteracts angiogenesis, which might be due to reduced NRP1-VEGFR2 mediated tip cell formation, or reduced leukocyte recruitment along with reduced growth factor supply and NET formation. As RNase A shows no signs of toxicity, but a high ribonucleolytic activity, it is reasonable to deduce that RNase A interferes with angiogenesis by degrading extracellular RNA. Together, this implicates a yet unrecognized role for extracellular RNA in angiogenesis. However, further studies are necessary to confirm this assumption as well as the proposed molecular mechanisms.

## Data Availability Statement

The original contributions presented in the study are included in the article/Supplementary Material and further inquiries can be directed to the corresponding author.

## Ethics Statement

The animal study was reviewed and approved by Bavarian Animal Care and Use Committee (Regierung Oberbayern; ethical approval code: ROB-55.2Vet-2532.Vet_02-17-99).

## Author’s Note

This article is dedicated to Klaus T. Preissner, a mentor, colleague, and meanwhile friend. Klaus is a liberal-minded and enthusiastic scientist always coming up with a lot of very fruitful and excellent ideas how to proceed in research. Klaus ich danke dir für all die herzerfrischenden Diskussionen. Es ist eine Freude mit dir zu arbeiten.

Lisa

## Author Contributions

ML and KK performed surgery. ML, KK, SK, SN, DH, and HI-A performed histological analyses. SM participated in scientific discussions. ML, HI-A, and ED wrote and revised the manuscript, designed the experiments, and analyzed the data. All authors contributed to the article and approved the submitted version.

### Conflict of Interest

The authors declare that the research was conducted in the absence of any commercial or financial relationships that could be construed as a potential conflict of interest.
