# Supplementary material for: RNase A Treatment Interferes With Leukocyte Recruitment, Neutrophil Extracellular Trap Formation, and Angiogenesis in Ischemic Muscle Tissue
Source: Front Physiol. 2020 Nov 6;11:576736. doi: 10.3389/fphys.2020.576736 (PMC7677187; doi:10.3389/fphys.2020.576736)
Supplement: Supplementary file 1 [file Table_1.pdf]

Mouse		Figure 2A tissue damage (%)
Saline 1	ischemic	7.8
Saline 2	ischemic	10.0
Saline 3	ischemic	16.2
Saline 4	ischemic	10.4
Saline 5	ischemic	13.0
RNase A 1	ischemic	31.3
RNase A 2	ischemic	18.7
RNase A 3	ischemic	32.5
RNase A 4	ischemic	27.7
RNase A 5	ischemic	27.2

Supplementary Table 1: Raw Data of Figure 2A
