# Supplementary material for: RNase A Treatment Interferes With Leukocyte Recruitment, Neutrophil Extracellular Trap Formation, and Angiogenesis in Ischemic Muscle Tissue
Source: Front Physiol. 2020 Nov 6;11:576736. doi: 10.3389/fphys.2020.576736 (PMC7677187; doi:10.3389/fphys.2020.576736)
Supplement: Supplementary file 2 [file Table_2.pdf]

Mouse		Figure 2C	Figure 3		Figure 4			Figure 5		Suppl. Figure 1
		$CD31^+/ACTA2^-$ cells (ratio per muscle fiber)	$CD45^+$ cells (per mm^2)	$Ly6G^+$ cells (per mm^2)	$CD68^+$ cells (per mm^2)	$CD68^+/MRC1^-$ cells (% of $CD68^+$)	$CD68^+/MRC1^+$ cells (% of $CD68^+$)	NETs expression (per mm^2)	MPO^+ cells (per mm^2)	$CD31^+/BrdU^+/ACTA2^-$ cells (ratio per muscle fiber)
Saline 1	ischemic	2.287	405	89	44	93	7	2.47	0.812	0.694
	non-ischemic	1.186	53	14	13			0.15	0.018	0.019
Saline 2	ischemic	2.831	460	108	43	88	12	3.60	0.844	0.942
	non-ischemic	1.370	55	17	11			0.29	0.022	0.025
Saline 3	ischemic	2.542	432	92	44	84	16	3.40	0.876	0.954
	non-ischemic	1.194	58	17	12			0.29	0.016	0.019
RNase A 1	ischemic	1.574	163	24	22	14	86	1.16	0.473	0.445
	non-ischemic	1.110	60	15	11			0.39	0.019	0.019
RNase A 2	ischemic	1.649	141	31	28	18	82	1.28	0.509	0.514
	non-ischemic	1.158	57	15	13			0.35	0.024	0.024
RNase A 3	ischemic	1.634	178	40	28	11	89	1.30	0.440	0.487
	non-ischemic	1.096	57	22	11			0.30	0.018	0.018

Supplementary Table 2: Raw Data of Figure 2 – Supplementary Figure 1
