# Supplementary figures and images for: RNase A Treatment Interferes With Leukocyte Recruitment, Neutrophil Extracellular Trap Formation, and Angiogenesis in Ischemic Muscle Tissue
Source: Front Physiol. 2020 Nov 6;11:576736. doi: 10.3389/fphys.2020.576736 (PMC7677187; doi:10.3389/fphys.2020.576736)

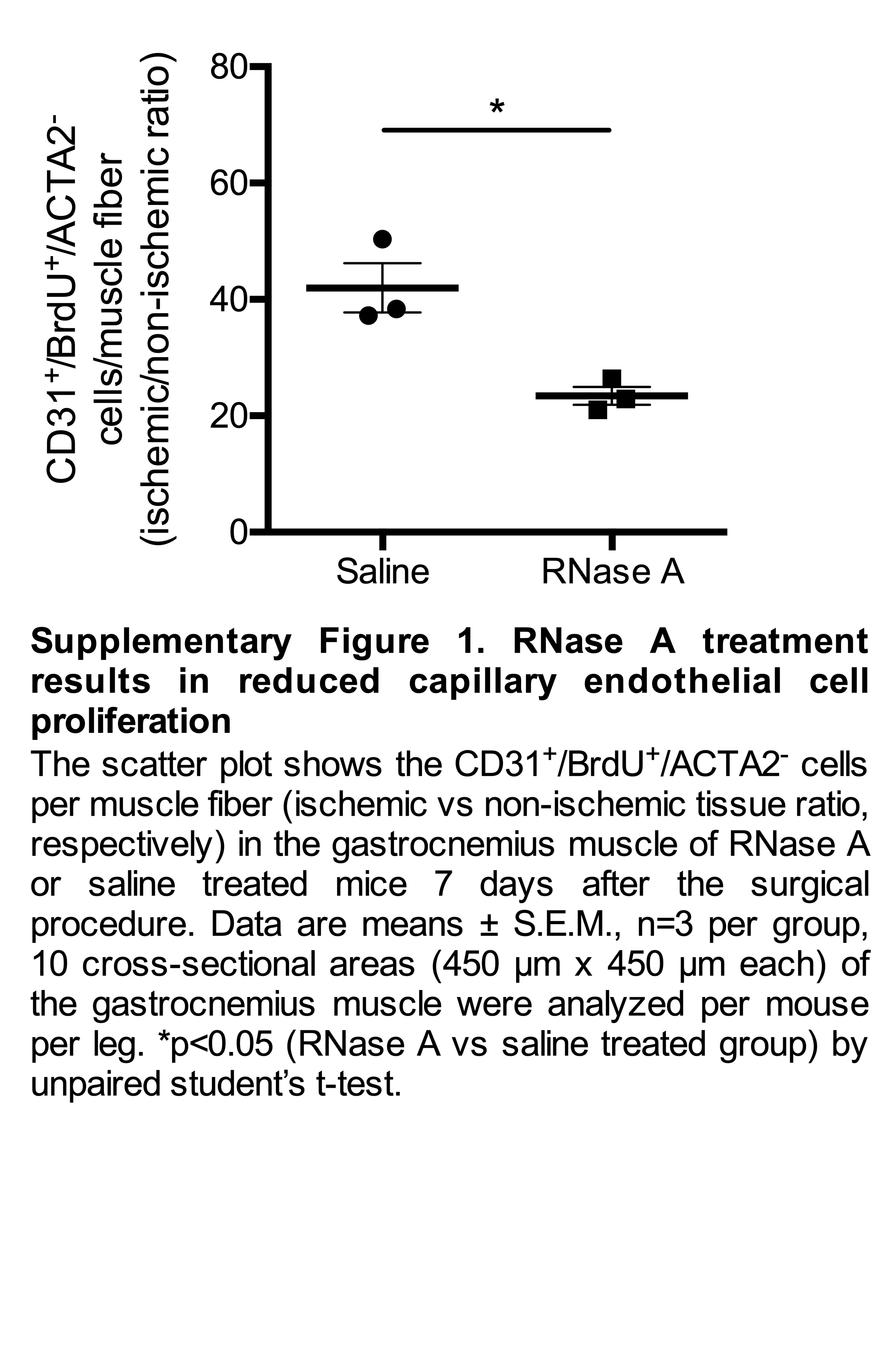

Supplement: Supplementary file 3 [file Image_1.tiff]

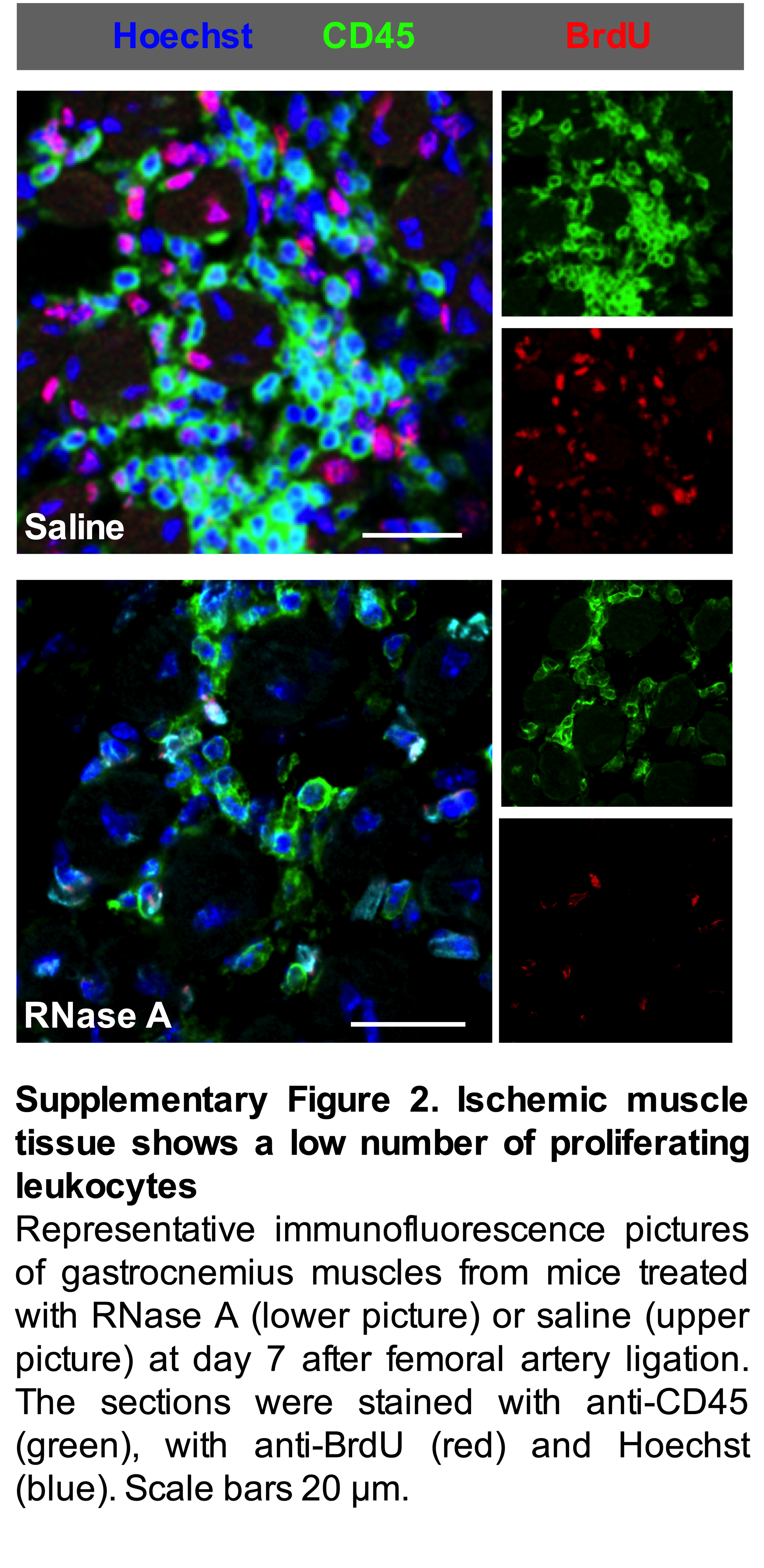

Supplement: Supplementary file 4 [file Image_2.tiff]
